# Continuous exercise induces airway epithelium damage while a matched-intensity and volume intermittent exercise does not

**DOI:** 10.1186/s12931-019-0978-1

**Published:** 2019-01-17

**Authors:** Adrien Combes, Jeanne Dekerle, Xavier Dumont, Rosie Twomey, Alfred Bernard, Frédéric Daussin, Valérie Bougault

**Affiliations:** 1 URePSSS, Unité de Recherche Pluridisciplinaire Sport, Santé, Société, Lille, France; 2 Fatigue and Exercise Laboratory, Centre for Sport Exercise Science and Medicine (SESAME), University of Brighton, Eastbourne, UK; 3 Louvain Center of Toxicology and Applied Pharmacology, Catholic University of Louvain, Brussels, Belgium; 4 LAMHESS, Université Côte d’Azur, Nice, France; 5 Eurasport, 413 Avenue Eugène Avinée, 59120 Loos, France

**Keywords:** Pneumoproteins, CC16, SP-D, Minute ventilation, Type of exercise

## Abstract

**Background:**

While continuous exercise (CE) induces greater ventilation ($\dot{V}$_E_) when compared to intermittent exercise (IE), little is known of the consequences on airway damage. Our aim was to investigate markers of epithelial cell damage – i.e. serum levels of CC16 and of the CC16/SP-D ratio - during and following a bout of CE and IE of matched work.

**Methods:**

Sixteen healthy young adults performed a 30-min continuous (CE) and a 60-min intermittent exercise (IE; 1-min work: 1-min rest) on separate occasions in a random order. Intensity was set at 70% of their maximum work rate (WR_max_). Heart rate (HR) and $\dot{V}$_E_ were measured throughout both tests. Blood samples were taken at rest, after the 10th min of the warm-up, at the end of both exercises, half way through IE (matched time but 50% work done for IE) as well as 30- and 60-min post-exercise. Lactate and CC16 and SP-D were determined.

**Results:**

Mean $\dot{V}$_E_ was higher for CE compared to IE (85 ± 17 l.min^− 1^ vs 50 ± 8 l.min^− 1^, respectively; *P* < 0.001). Serum-based markers of epithelial cell damage remained unchanged during IE. Interaction of test × time was observed for SP-D (*P = 0.02*), CC16 (μg.l^− 1^) (*P = 0.006*) and CC16/SP-D ratio (*P = 0.03*). Maximum delta CC16/SP-D was significantly correlated with mean $\dot{V}$_E_ sustained (*r* = 0.83, *P* < 0.001) during CE but not during IE.

**Conclusion:**

The 30-min CE performed at 70% WR_max_ induced mild airway damage, while a time- or work-matched IE did not. The extent of the damage during CE was associated with the higher ventilation rate.

## Background

A wealth of research has been today published on the potential additional benefits of intermittent exercise (IE) when compared to continuous exercise (CE) on key discriminants of respiratory, cardiovascular, and muscular function in both healthy and clinical populations [1–7]. Studies have yet to focus on the integrity of the airways. Some exercises may exacerbate airway inflammation and the loss of epithelium integrity in patients with respiratory disease and the long-term consequences on the airways are yet to be determined. The general consensus is in favour of the use of IE for health [1, 6–8]. A few studies recently suggested that IE may allow individuals to exercise at higher intensities longer, but that this may not be the sole stimulus for the health benefits of IE [8, 9]. Interestingly, it has also recently been recognised that there may not be such a need to exercise at high intensities when using intermittent training, and that moderate-intensity intermittent exercise may also be of benefit in regards to specific health outcomes [9]. In comparison to high-intensity interval training (HIIT), moderate-intensity interval training (MIIT) may be more appealing and/or feasible in untrained adults, clinical populations and/or those with increased risk of cardiovascular events [9]. Due to the lower levels of ventilation, MIIT is more likely to preserve the integrity of the airways in populations suffering from respiratory disease that are characterized by chronic airway inflammation and remodeling.

In particular, high intensity exercise sustained for several minutes may induce significant airway inflammation and/or trigger exercise-induced bronchoconstriction or asthma [1, 10, 11]. This transient inflammation may then aggravate existent airway pathologies, with the extent of exercise/hyperpnea-induced airway inflammation dependent upon EIB/asthma status [12–16]. However, epithelial shedding is induced by high levels of ventilation (> 60% of maximal voluntary ventilation) and is not necessarily associated with the presence of bronchoconstriction [16–18]. Therefore, high levels of ventilation may induce airway damage even in healthy participants [16–20]. As for EIB, the underlining mechanisms are thought to be dehydration of the airways and probably the mechanical stress applied to the epithelium during hyperventilation [21]. The higher ventilation rate with continuous exercise (CE) for the same intensity of exercise may bring along greater stress onto the epithelium cells of the airways. If there is evidence of less damage to the epithelium with IE, this may be a reason to recommend IE over CE in some practical settings.

Serum levels of lung proteins such as the anti-inflammatory club cells protein 16kD (CC16) secreted in the airways and alveolar surfactant-associated serum proteins (SP-A, SP-B, and SP-D) are used as markers of lung epithelial barrier integrity in a variety of acute or chronic lung disorders [22]. The serum CC16/SP-D ratio is considered to be one of the most valid and sensitive marker for lung epithelium damage in the case of toxicant or irritant inhalation [22]. Surprisingly, to our knowledge, no study has investigated the effect of the type of exercise – that is continuous vs intermittent exercise - on these markers of epithelial damage. Investigating these two types of exercise may provide valuable insight into the role of ventilation on the development of airway damage.

The purpose of the present study was therefore to investigate gold standard markers of epithelial cell damage – i.e. serum levels of CC16 and CC16/SP-D ratio - during and following a bout of CE and IE of matched work. It was hypothesized that the IE would be characterized by lower ventilation rate ($\dot{V}$_E_) because of the inherent nature of the exercise (i.e. intercepted periods of active recovery) and lower levels of CC16 and CC16/SP-D ratio, indicating lesser airway damage.

## Materials and methods

### Participants and ethical considerations

Recreationally active males (*n* = 16) participated in the study. Participants were instructed to refrain from physical activity, alcohol and caffeine for ≥48 h prior to testing sessions. Volunteers under medical treatment for asthma (daily inhaled corticosteroids and/or beta_2_ agonists) were excluded. All the participants provided written informed consent prior to participation. The protocol was approved by the University of Brighton Ethics Committee, and the study was conducted according to the 2008 version of the Declaration of Helsinki.

### Study design

A prior screening of the volunteers ensured inclusion and exclusion criteria were met. All eligible participants then visited the laboratories for three separate visits to perform (1) an incremental, (2) a continuous, and (3) an intermittent exercise. All visits were separated by ≥48 h and the order of the experimental trials (2 and 3) was randomised. Both CE and IE were scheduled at the same hour of the day and conducted in consistent environmental conditions (Temperature: 19 ± 1 °C and humidity: 55 ± 3%). Heart rate (HR; RS 800, Polar, Kempele, Finland) and gas exchange (MediSoft, Germany) were measured throughout exercise. Volume and gas calibrations for the breath-by-breath measurements were performed before each test.

### Incremental exercise test

Participants completed an incremental exercise test on an electrically braked ergometer (SRM, SRM GmbH, Germany) to determine maximal oxygen uptake ($\dot{V}$O_2max_) and maximum work rate (WR_max_). After a 3-min warm-up period at 75 W, work rate was increased by 25 W every 2 min until volitional exhaustion. Each participant carried out a maximal effort [23]. $\dot{V}$O_2max_ was defined as the highest 30-s average of breath-by breath $\dot{V}$O_2_. WR_max_ corresponded to the highest 60-s average work rate. Theoretical maximum heart rate (HRt_max_) was calculated as 220-age (years). Gas exchange threshold (GET) was determined independently by two experienced physiologists. GET was determined using visual inspection from a cluster of measures including: 1) the first disproportionate increase in carbon dioxide output ($\dot{V}$CO_2_) from individual plots of $\dot{V}$CO_2_ versus $\dot{V}$O_2_; 2) an increase in $\dot{V}$E/$\dot{V}$O_2_ with no increase in $\dot{V}$E /$\dot{V}$CO_2_ [24]; and 3) the first break (increase) in the evolution in $\dot{V}$E versus time [25].

### Continuous and intermittent exercises

Both CE and IE were performed on a customised cycle ergometer (620 Ergomedic; Monark, Varberg, Sweden) fitted with power measuring cranks (Pro Track, 8; SRM). A standardised 10-min warm-up performed at 40% WR_max_ was followed with a 5-min resting period prior to the start of the exercise bout. For CE, the exercise involved cycling for 30 min at a continuous intensity of 70% WR_max._ For IE, the exercise involved intervals of 1 min cycling at 70% WR_max_ interspersed with a 1-min passive recovery (seated on the ergometer). The 1-min work:1-min rest was repeated 30 times, such that the total duration of the trial was 60 min, but the work done was matched to the CE trial.

Minute averages were calculated from the breath-by-breath and heart rate measurements. For both conditions, mean minute ventilation ($\dot{V}$_E_) and overall volume of air ventilated (30 × mean $\dot{V}$_E_ for CE and 60 × mean $\dot{V}$_E_ for CE) were calculated. Flow-volume loops were obtained using a portable spirometer (Easy One, Dyn’R, France) before and 5-min after each test to check for the absence of exercise-induced bronchoconstriction. Two reproducible measurements of forced expiratory volume in one second (FEV_1_) and forced vital capacity (FVC) were obtained at these time-points. The theoretical maximal voluntary ventilation (MVVt) was calculated as 35 times the baseline FEV_1_ for each participant [26].

### Serum samplings

Blood samples (10 mL per sample) were taken before, during and twice after exercise. After the baseline spirometry test, a venous catheter was placed in a brachial vein to allow for repeated sampling. Blood was sampled at the end of both exercises (30 min for CE, 60 min for IE; matched work done), half way through IE (matched time but 50% work done for IE) as well as 30- and 60-min post-exercise. Blood was then sampled into dry tubes and left in a standing position for ~ 2 h to let the clot to form. The blood was then centrifuged at 4 °C, 3000 rpm for 10 min, and micro-tubes were stored at − 80 °C until analysis. Additionally, for each time point, blood lactate concentration ([La]) was determined immediately using a Yellow Springs Instrument (YSI 2300 Stat Plus; Analox, Sheffield, UK). The analyser was calibrated regularly using precision standards and regularly evaluated by external quality controls.

### Serum CC16 and SP-D analyses

Club cell proteins (CC16) were measured by an automated latex immunoassay, which was validated by comparison with a fluorescence enzyme immunoassay using monoclonal antibodies [27]. The serum concentration of SP-D was determined using a commercially available ELISA kit (code no. YSE-7744; Yamasa Corporation, Choshi, Japan). Serum creatinine was quantified by the Beckman Synchron CX5 Delta Clinical System (Beckman Coulter Inc., Fullerton, CA, USA). Serum raw data for CC16 and SP-D were expressed as μg.l^− 1^. Serum CC16 was also expressed as ng.mg^− 1^ creatinine (weighted CC16) and as CC16/SP-D. All the measures were performed in duplicate by the same person who was blinded to the coded samples (X.D.).

### Statistical analyses

Data was analysed using SigmaStat (Software version 3.5). The univariate normality and homogeneity of variance were verified with the Shapiro-Wilk test and Brown–Forsythe variation of Levene’s test, respectively. Paired-sample *t*-tests were performed to test for differences in work rate, accumulated work done, mean $\dot{V}$_E,_ overall volume of air ventilated, maximum deltas CC16, CC16/SP-D ratio and SP-D between CE and IE. Two-way ANOVAs with repeated measures were carried out to identify significant differences in CC16, SP-D, CC16/SP-D ratio, spirometric values, HR, $\dot{V}$_E,_ breathing frequency (Bf) and tidal volume (VT) over time and between the two tests. Serum CC16 and CC16/SP-D were analysed using log-transformed values and SP-D using square root-transformed values, to meet the normality assumption. The Holm-Sidak post hoc test for multiple comparisons was applied to localize any differences. Delta changes from warm-up were calculated as the difference between the maximum value post-exercise and the warm-up value, divided by the warm-up value. Bivariate correlations between delta change in CC16, SP-D, and CC16/SP-D, and ventilation-based variables were analysed using the Pearson correlation coefficient (*r*). A *P* value < 0.05 was considered statistically significant. Data is presented as mean ± SD.

## Results

Twenty-one participants were recruited. Due to illness or technical problems, five participants were excluded from the analysis. The remaining 16 participants’ characteristics are presented in Table 1. The mean work rate for CE and IE were not significantly different (200 ± 26 W vs 199 ± 26 W, respectively, *t = 0.91*, *P* = 0.38), and corresponded to 101 ± 7% of GET.

**Table 1:** Participants' characteristics and maximal values to incremental exercise test (*n* = 16)

Characteristic	Mean ± SD
Age (years)	24 ± 5
Height (cm)	180 ± 7
Mass (kg)	74 ± 10
Body Mass Index (kg.m^−2^)	23 ± 2
FEV_1_ (% pred)	104 ± 10
FVC (% pred)	105 ± 12
VO_2max_ (ml.kg^− 1^.min^− 1^)	45.4 ± 7.4
WR_max_ (W)	280 ± 35
V_Emax_ (l.min^− 1^)	161 ± 26
V_Emax_ (% MVVt)	93 ± 13
Bf_max_ (breaths.min^− 1^)	55 ± 10
VT_max_ (l)	2.79 ± 0.52
VT_max_ (% FVC)	50 ± 6
HR_max_ (beats.min^−1^)	186 ± 11
HR_max_ (%HRt_max_)	95 ± 6
Lactate concentration max (mmol.l^−1^)	8.6 ± 1.7

FEV_1_: Forced expiratory volume in one second; FVC: forced vital capacity; VO_2_ max: maximal oxygen uptake; WR_max_: incremental maximum work rate, V_Emax_: maximal voluntary ventilation, MVVt: theoretical maximal voluntary ventilation = 35 × FEV_1_; Bf: breathing frequency; VT: tidal volume; HR: Heart rate; HRt_max_: theoretical maximal heart rate (220-age)

**Fig. 1:**
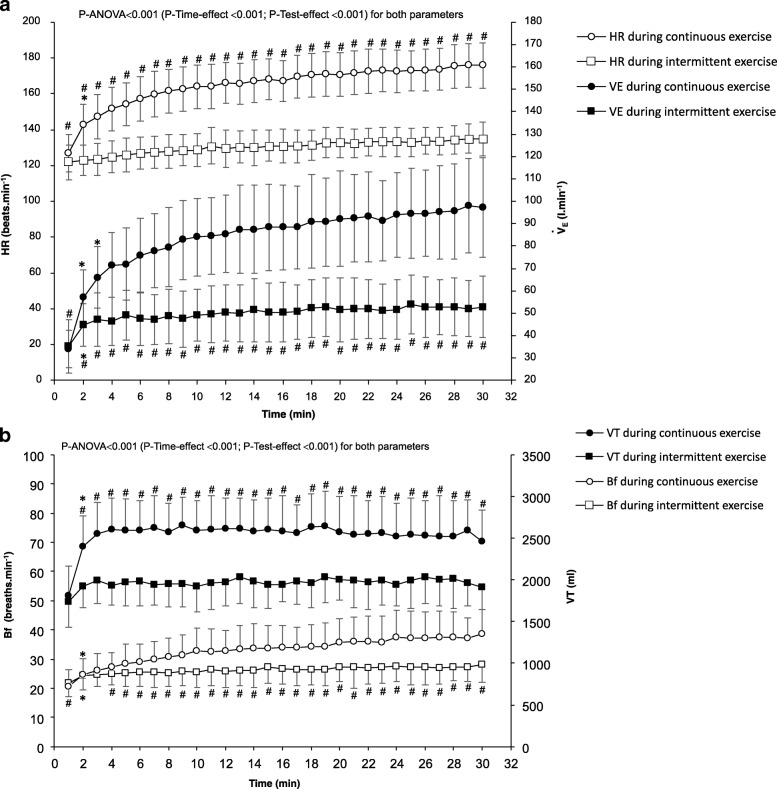
Evolution during the 30-min work periods of continuous and intermittent exercises of heart rate and ventilation (a), and tidal volume and breathing frequency (b). Parameters are expressed as mean and standard deviations at each minute of the tests. HR: heart rate; $\dot{V}$_E_: ventilation; Bf: breathing frequency: VT: tidal volume. For intermittent exercise lasting one hour, only data from the 30 min of work were analyzed (recovery periods have not been taken into account into the graph). ^*^Post-hoc *P* < 0.05 between a time-point and the previous one during a same test; ^#^Post-hoc P < 0.05 between both tests at a same time-point

**Fig. 2:**
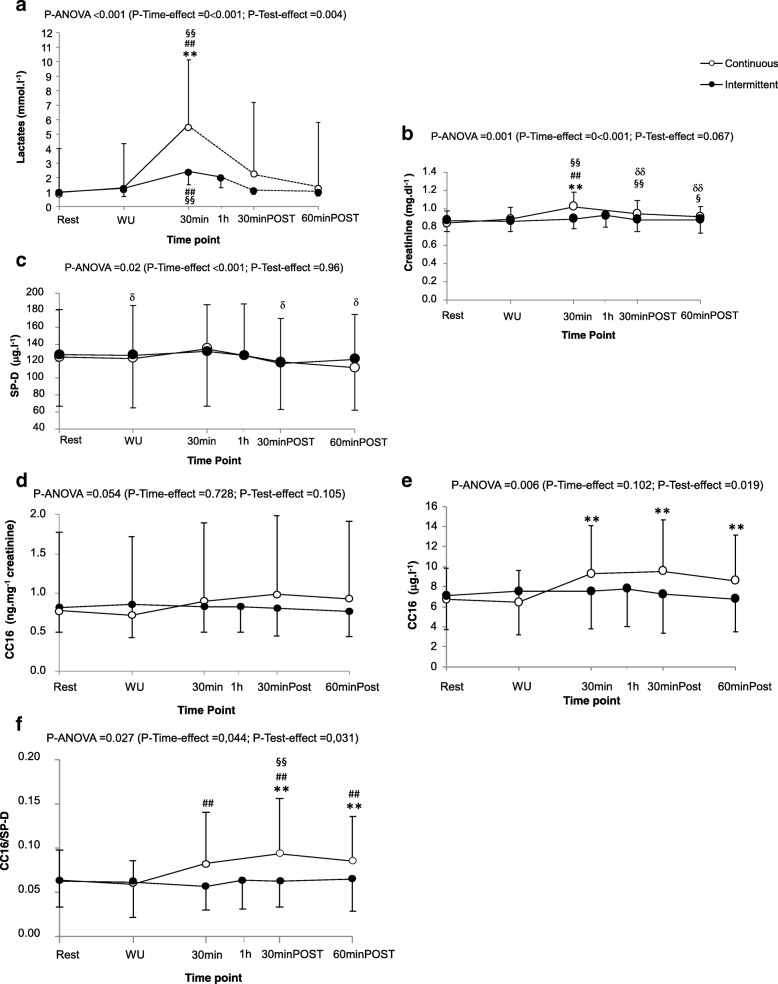
Evolution of blood lactate (a), creatinine (b), SP-D (c), serum values of CC16 (d and e), and CC16/SP-D ratio (f). Data are presented as mean values±SDs. WU: serum sampled at the end of the 10-min warm-up; 30 min: serum sampled at 30-min of exercise; 1 h: end of intermittent exercise; 30-minPOST and 60-minPOST: serum sampled 30-min and 60-min after the end of exercise, respectively. (a) Only 7 participants had serum lactate during 30-minPOST and 60-minPOST, therefore the ANOVA was performed only on rest data, and warm-up and end of exercise (full line). For information, 30-minPOST and 60-minPOST were also represented (Dotted line). ^*^ and ^**^Post-hoc P < 0.05 and 0.01, respectively, between both tests at a same time-point. ^##^ post-hoc *P* < 0.01 compared with warm-up; ^§§^ post-hoc *P* < 0.01 compared with rest; ^δ^post-hoc *P* < 0.05 compared with 30 min value

Changes in $\dot{V}$_E_ and HR are presented in Fig. 1a for both exercises. Mean HR sustained during cycling was significantly higher for CE (90 ± 9% of HR_max_; IE: 70 ± 2% of HR_max_; *t = 20,14, P* < 0.001). Mean $\dot{V}$_E_ during the cycling was also significantly higher for CE (85 ± 17 l.min^− 1^ vs 50 ± 8 l.min^− 1^ for IE; *t = 13.69, P < 0.001*). For CE, mean $\dot{V}$_E_ was 57 ± 8% of $\dot{V}$_Emax_, 53 ± 12% of MVVt and 134 ± 17% of $\dot{V}$_E_ at GET. These values were lower for IE, where mean $\dot{V}$_E_ was 34 ± 4% of $\dot{V}$_Emax_, 31 ± 6% of MVVt and 79 ± 10% of $\dot{V}$_E_ at GET (all *P* < 0.001 vs. CE). The mean $\dot{V}$_E_ during the 30-min continuous exercise was systematically above the $\dot{V}$_E_ corresponding to GET; whereas this was the case for only one participant for IE. Changes in VT and Bf are presented Fig. 1b. Mean VT during the 30 min of exercise represented 46 ± 4% and 35 ± 4% of resting FVC for CE and IE, respectively (*t = 10.72, P* < 0.001). For CE, mean VT was 94 ± 15% of VT_max_ and 110 ± 20% of VT at GET. In comparison, mean VT was 72 ± 13% of VT_max_ and 84 ± 13% of VT at GET for IE (both *P* < 0.001 vs. CE). Mean Bf was 62 ± 11% of Bf_max_ or 124 ± 16% of Bf at GET for CE, and 49 ± 9% of Bf_max_, or 97 ± 13% of Bf at GET for IE (both *P* < 0.001). No significant difference was observed for FEV_1_ at any time-point between the two exercises (test × temps interaction: *F = 0.71*, *P* = 0.53). Blood lactate concentrations were significantly different at the end of exercise with higher values for CE (5.3 ± 1.9 mmol.l^− 1^) when compared to IE (2.0 ± 0.7 mmol.l^− 1^; *P* < 0.001) (test × temps interaction: *F = 37.5*, *P* < 0.001) (Fig. 2a).

There was no difference of serum creatinine between both tests (*F = 3.91, P = 0.07*), but a significant effect of time (*F = 12.62, P < 0.001*) and an interaction of test × time (*F = 5.20, P = 0.001*). It increased at the end of exercise, 30-min and 60-min post-exercise compared with baseline, but for CE only (*P < 0.05* for the three time-points) (Fig. 2b). For serum SP-D, there was no difference between the two tests (*F = 0.02, P = 0.96*), but a significant effect of time (*F = 5.42, P < 0.001*) and an interaction of test × time (*F = 3.18, P = 0.02*) (Fig. 2c). Values at the end of CE were significantly higher compared with warm-up, 30-min and 60-min post-exercise (*P* < 0.01 for the three time-points). For serum CC16 weighted by creatinine, there was no significant difference between the two tests (*F* = 2.95, *P* = 0.11), or over time (*F* = 0.51, *P* = 0.73), and no test × time interaction (*F* = 2.9, *P* = 0.054) (Fig. 2d). When CC16 was expressed as μg.l^− 1^ (not weighted by creatinine), a test effect was observed (*F = 6.7, P = 0.02*), but no effect of time (*F = 2.02, P = 0.10*), and a significant test × time interaction (*F = 4.0, P = 0.006*) (Fig. 2e). For CC16/SP-D ratio, a significant difference between tests was observed (*F* = 5.56, *P* = 0.031), with a time effect (F = 2.60, *P* = 0.044) and an interaction of test × time (*F* = 2.98, *P* = 0.027) (Fig. 2f). The values were significantly higher at 30-min and 60-min post-exercise after CE (*P* = 0.005 and *P* = 0.007, respectively). A significant difference was observed in maximum delta CC16/SP-D ratio between both exercises (*t = 2.30, P* = 0.036) but not for CC16 (*t* = 2.09, *P* = 0.054) or SP-D *(t = 0.05, P = 0.96*).

**Fig. 3:**
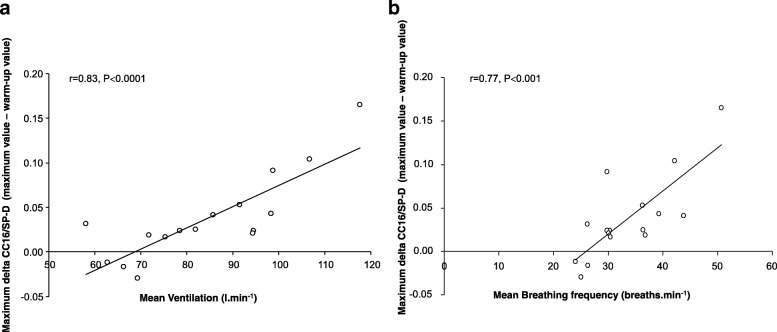
Relationship between the maximum delta CC16/SP-D ratio and (a) the mean ventilation during CE, and (b) the mean breathing frequency during CE. The maximum delta value was calculated by subtracting each participants’ maximum value during or after continuous exercise and his end-warm-up value

Strong correlations were observed between mean $\dot{V}$_E_ sustained during CE (whether expressed as l.min^− 1^ or overall volume of air ventilated during the time period) and markers of epithelial cell damage (maximum delta CC16/SP-D: *r* = 0.83, *P* < 0.001, Fig. 3a; maximum delta CC16: *r* = 0.65, *P* = 0.006). Mean Bf during CE also correlated to maximum delta CC16/SP-D (*r* = 0.77, *P* < 0.001, Fig. 3b) and maximum delta CC16 (*r* = 0.59, *P* = 0.01).

## Discussion

The present study demonstrates that 30 min of CE performed at an intensity of 70% of WR_max_, corresponding approximately to the intensity of the GET, induces more airway damage than the same work done in 60 min of IE (1 min work / 1 min rest) in healthy young participants. Positive bivariate correlations between the main markers of the epithelial cell damage and ventilation-based variables suggest for the extent of the damage to be associated with the greater ventilation rate sustained during the CE. Conversely, IE did not induce any change in any of the serum-based measures leading to the conclusion that the reduced ventilation rate as a consequence of the regular periods of recovery in this type of exercise may sufficiently reduce stresses on the lung airways so that airway damage is prevented.

Previous studies have reported either an increase or no change in CC16 following various types of exercise with greater chance of effect when exposed to chlorine or ozone [19, 28–32]. Discrepancies in the literature may be due to the wide range of exercise intensity, duration, and modalities. Exercise intensity may indeed play a determinant role in CC16 changes, with greater changes at higher intensities [33]. A bout of exercise at 60–75% of maximum HR and maintained for 1.5 or 2 h did not induce any change in CC16, whether immediately or several hours’ post-exercise [29, 30], while a more intense exercise (HR > 75–80% HR_max_) sustained during shorter duration (6 min to 1.5 h) was shown to increase urine or blood CC16 immediately post-exercise [16, 20, 34, 35], with a peak value 30 min to 1 h after the end of the exercise [16]. In agreement with the literature, the present findings demonstrate greater serum-based markers of epithelium cell damage after 30 min of CE (88 ± 6% of HR_max_), thus immediately, 30 and 60 min post-exercise. The findings from all of these studies suggest that above 75–80% of HR_max_, the greater the exercise intensity, the greater the release of CC16.

Minute ventilation may have to be above a certain threshold for any change in CC16 to be observed, thus irrespective of the duration of the exercise [18, 33]. The significance in correlation between markers of airway damage and ventilation-based variables in the present study corroborates this viewpoint. We observed a strong bivariate correlation between $\dot{V}$_E_ sustained during CE and CC16/SPD ratio. The breathing pattern with high Bf and VT during CE when compared to IE, may be leading to dehydration and mechanical damage of the airway. In a review on airway injury in endurance sport athletes [21], the authors underlined the role of exercise hyperpnoea on the epithelial layer disruption. High ventilation rate would induce a mechanical stress through the stretching of epithelial tight junctions and high-forces generated on the epithelium that increases transpithelial pressure gradients, and the dehydration of the airways, with a consequent change in tonicity, and viscosity of the airway surface liquid [21]. The lack of significant changes in serum CC16 and CC16/SP-D ratio after IE in our study also supports the requirement for elevated exercise ventilation rate for CC16 changes to be observed, and so airway damage to be induced. The correlation of Bf with markers of epithelial damage are also in line with the postulate that breathing frequency is a determinant factor that must be considered when considering airway damage under exposure to inhaled pollutants [29].

The present results may be interesting in the context of pathologies and particularly in patients with uncontrolled asthma or exercise-induced asthma. The airway response to exercise of asthmatic populations may vary depending upon the exercise design, with high inter-individual differences. With exercise-induced asthma, it is a question of balance between bronchoconstriction and bronchodilation mechanisms [36]. During CE at a moderate intensity, in asthmatics having exercise-induced bronchoconstriction (EIB) but with normal lung function at rest, after a possible short bronchodilation at the very beginning of the exercise, no change in airway calibre or a slight bronchoconstriction occurs, before the post-exercise fall in FEV_1_ [37]. During IE, if the periods of work are long (i.e. 4 to 6 min) and of moderate to high intensity, a bronchodilation during the working period is followed by a constriction during the resting period with FEV_1_ falling post-exercise. This is similar to what is observed after a continuous exercise [37]. A recent study showed among 5 mild asthmatics having EIB after a 20 min CE (65% WR_max_), that only 2 developed EIB after short work period duration of high intensity IE (10 times [1 min work at 90% WR_max_ / 1 min rest et 10% WR_max_]), and none after a moderate intensity IE (10 times [1 min work at 65% WR_max_ / 1 min rest et 10% WR_max_]) [38]. High intensities IE in intermittent mild asthmatics may trigger EIB in those having a quite severe fall in FEV_1_ post-exercise (> 30%), probably due to the high level of ventilation during the work period [38]. In these patients, it could be recommended to perform IE at lower intensities (70% WR_max_ or GET). This would reduce the chance of developing EIB because of the lessened airway dehydration and mechanical stress, as evidenced by the absence of ventilation-induced epithelial damage in the present study.

A slight limitation to the present study is the absence of direct measurement of airway damage through quantification of the number of bronchial epithelial cells in induced sputum during both types of exercise. While pneumoproteins are used as biomarkers of epithelial damage [5, 16–20], no study has validated their measurement in blood or urine for a direct marker of epithelial damage after exercise or hyperventilation. In healthy young subjects, a sufficient quantity of induced sputum for analysis may be difficult to obtain; indeed, a previous study reported a successful rate of only 40% for one sample [39].

## Conclusions

To conclude, we observed an increase in serum airway damage biomarkers after a 30-min continuous exercise performed at an intensity of 70% WR_max_, while no significant change was observed during or after a work-matched intermittent exercise performed at a same intensity. The level of epithelium cell damage was also positively related to ventilation rate; breathing frequency more specifically. We proposed the idea of a ventilation cut-off or threshold that would need to be exceeded for airway damage to be observed. Amateur and professional endurance competitive athletes often use intermittent exercise so that to increase the intensity of training that can be sustained over time. While in the present study, exercise intensity was relatively low for the intermittent condition, compared to more traditional training intensities, as its control allowed us to discriminate the sole effect of the exercise modality on airway damage, furthers studies may be conducted to determine whether intermittent exercise of higher intensity may also induce airway damage, and if so, whether there is an optimum work: rest ratio that would facilitate the protection of the airways.

## Abbreviations

Bf: breathing frequency
CC16: Club cells protein 16kD
CE: continuous exercise
EIB: exercise-induced bronchoconstriction
FEV_1_: forced expiratory volume in one second
FVC: forced vital capacity
GET: Gas exchange threshold
HIIT: high –intensity interval training
HR: heart rate
HRt: theorical heart rate
IE: intermittent exercise
MIIT: moderate-intensity interval training
MVVt: theorical maximum voluntary ventilation
SP-D: surfactant associated protein D
$\dot{V}$CO_2_: carbon dioxide output
$$ \dot{V} $$_E_: ventilation rate
$$ \dot{V} $$O_2_: oxygen uptake
VT: tidal volume
WR: work rate
